# Histology of metastatic colorectal cancer in a lymph node

**DOI:** 10.1371/journal.pone.0284536

**Published:** 2023-04-13

**Authors:** Shozo Yokoyama, Takashi Watanabe, Yoichi Fujita, Shuichi Matsumura, Katsuya Ueda, Shotaro Nagano, Ikuharu Kinoshita, Daisuke Murakami, Hirotaka Tabata, Toshiaki Tsuji, Satoru Ozawa, Takuya Tamaki, Yoshihiro Nakatani, Masami Oka

**Affiliations:** Department of Surgery, National Hospital Organization Minami Wakayama Medical Center, Tanabe, Japan; Kimura Hoospital, JAPAN

## Abstract

**Background:**

A primary colorectal cancer (CRC) tumor can contain heterogeneous cancer cells. As clones of cells with different properties metastasize to lymph nodes (LNs), they could show different morphologies. Cancer histologies in LNs of CRC remains to be described.

**Methods:**

Our study enrolled 318 consecutive patients with CRC who underwent primary tumor resection with lymph node dissection between January 2011 and June 2016. 119 (37.4%) patients who had metastatic LNs (mLNs) were finally included in this study. Cancer histologies in LNs were classified and compared with pathologically diagnosed differentiation in the primary lesion. The association between histologies in lymph node metastasis (LNM) and prognosis in patients with CRC was investigated.

**Results:**

The histologies of the cancer cells in the mLNs were classified into four types: tubular, cribriform, poorly differentiated, and mucinous. Same degree of pathologically diagnosed differentiation in the primary tumor produced various histological types in LNM. In Kaplan–Meier analysis, prognosis was worse in CRC patients with moderately differentiated adenocarcinoma who had at least some mLN also showing cribriform carcinoma than for those whose mLNs all showed tubular carcinoma.

**Conclusions:**

Histology in LNM from CRC might indicate the heterogeneity and malignant phenotype of the disease.

## Introduction

Recognizing tumor heterogeneity is important in cancer therapy [1–3]. In tumor heterogeneity, the primary tumor contains various cancer cell clones, some of which sometimes resist various treatments. Cancer cells can metastasize as single cells or clusters, proliferating and organizing at the metastatic site according to their clonal histology. In colon cancer, lymph nodes metastasis (LNM) has been reported to be polyclonal at the genetic level [4], and thus is hypothesized to be heterogeneous. As a result, morphologic changes might depend on the histologic properties of the clones.

Lymph node metastasis is a prognostic factor for patients with colorectal cancer (CRC). Adjuvant chemotherapy for stage III CRC is based on nodal metastasis and is standardized worldwide [5], being an oxaliplatin-based chemotherapy such as FOLFOX or XELOX for 6 months [6,7]. The LNM are therefore a suitable target for evaluating CRC phenotypes. The current TNM staging paradigm uses only the number of metastatic lymph node (mLN), and not their cancer histology. We reported that at least 1 mLN showing cribriform carcinoma is associated with distant metastasis in patients with node-positive CRC and is correlated with recurrence and survival in stage III disease [8].

In the present study, we set out to detail the cancer histology in LNM from CRC, any similarity in the degree of differentiation between the LNM and the primary tumor, and the suitability of cancer histology in the mLNs for prognosticating the cancer-specific survival (CSS) of patients with node-positive CRC.

## Methods

### Patients

The study enrolled 318 consecutive patients with CRC who underwent colectomy, anterior resection, abdominoperineal resection, or Hartmann operation with lymphadenectomy between January 2011 and June 2016 at the National Hospital Organization Minami Wakayama Medical Center (Tanabe, Japan). The follow-up period was 5 years. The Ethics Committee of National Hospital Organization Minami Wakayama Medical Center approved the study (#2021–10). Medical data records were accessed for the purpose of this study after the ethics committee approval on January 24^th^, 2021. All methods were performed in compliance with the Declaration of Helsinki, the guidelines for ethical principles for medical research involving human subjects, and the ethics guidelines of the National Hospital Organization Minami Wakayama Medical Center. Informed consent was obtained in the form of opt-out on the web page of the National Hospital Organization Minami Wakayama Medical Center.

### Histological analysis

The hematoxylin and eosin stained colorectal and LN tissue sections were examined microscopically. All specimens were blindly reviewed twice by three individuals (SY, TW and YF). Where discrepancies arose, these specimens were discussed to achieve a consensus. All pictures were acquired using an Olympus CX33 (Olympus, Japan) with NY1S adaptor (Micronet, Japan), EOS X9 and EOS utility software program (Canon, Japan). All mLNs were assessed to evaluate the histological type.

### Statistical analyses

The Kaplan–Meier method was used to estimate postoperative survival, and a log-rank test was used to determine statistical significance. A *p* value less than 0.05 was considered statistically significant. All calculations were performed using the JMP Pro software application (version 14.1.0: SAS Institute, Cary, NC, USA).

## Results

### Patient characteristics

Of 318 patients with CRC, 119 (37.4%) patients who had LNM were finally included in this study. Using the Union for International Cancer Control TNM classification, staging was III for 87 patients and IV for 32 patients. Mean age in this cohort was 70 years (range: 34–89 years), there were 67 males and 52 females. The resected tumors were located in either the colon (*n* = 74) or the rectum (*n* = 45). The number of patients with T stage: T2, T3, T4a and T4b are 4, 84, 30, and 1, respectively. Patients with stage III tumors received 5-fluorouracil–based postoperative chemotherapy. Of 32 patients stage IV tumors, 6 (17.1%) patients underwent R0 resection. The average of the number of LNs harvested was 15.7. The average of the number of mLNs was 4.3.

### Cancer histology in mLNs from CRC

**Fig 1:**
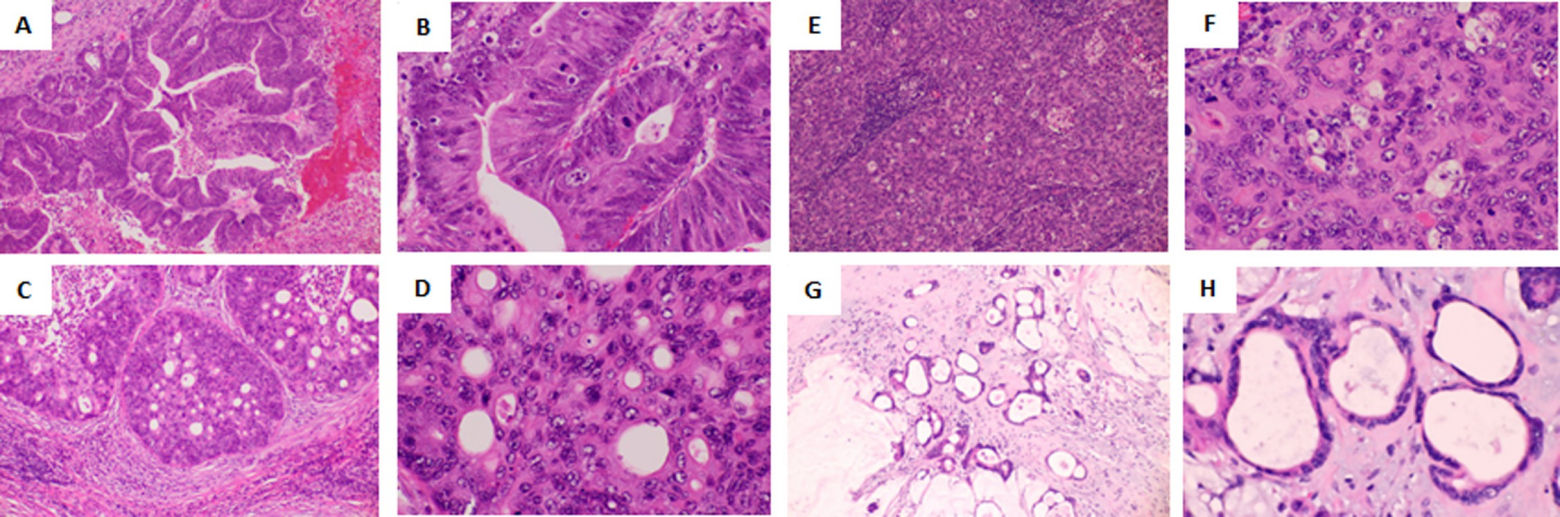
Cancer histology in metastatic lymph nodes. (A,B) Tubular. (C,D) Cribriform. (E,F) Poorly differentiated. (G,H) Mucinous. Original magnification: A, C, E, G: 100×; B, D, F, H: 400×. Staining: Hematoxylin and eosin.

**Fig 2:**
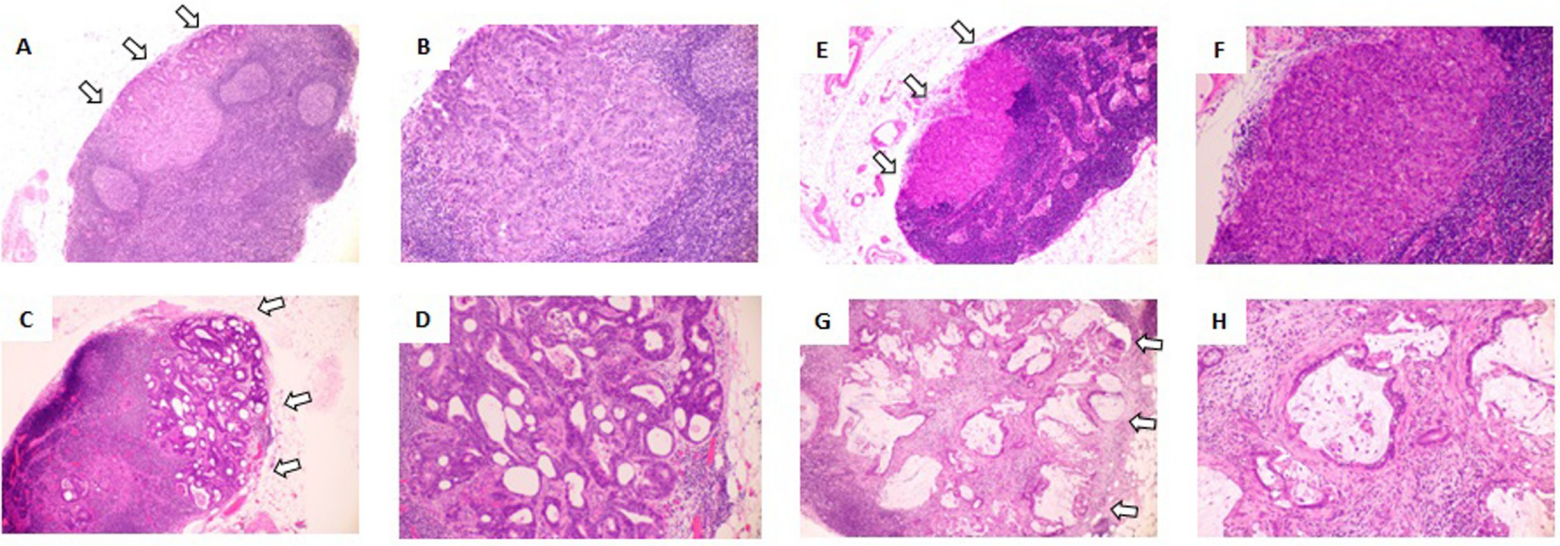
Homogeneous cancer histology presenting in a single metastatic lymph node. (A,B) Tubular adenocarcinoma. (C,D) Cribriform adenocarcinoma. (E,F) Poorly differentiated adenocarcinoma. (G,H) Mucinous adenocarcinoma. Original magnification: A, C, E, G: 40×; B, D, F, H: 100×. Staining: Hematoxylin and eosin.

We first defined the cancer histology types observed in LNM from CRC. Because adenocarcinomas form ducts, we based the classification on the shape of the hollow cavities and on the cellular polarity, focusing especially on whether the cavities were slender and elongated or rounded hollows. “Tubular” was defined as an elongated hollow cavity with cancer cell polarity (Fig 1A and 1B). “Cribriform” was defined as small rounded cavities with no cancer cell polarity (Fig 1C and 1D). “Poorly differentiated” was defined as having almost no hollow cavities and no cancer cell polarity (Fig 1E and 1F). “Mucinous” was defined as containing mucin in the cavity (Fig 1G and 1H). In this study, each mLN included a single histologic type growing as a mass, but not multiple histologic types (Fig 2A–2H). Based on the histological classification in LNM, tubular, cribriform, poorly differentiated, or mucinous type, the morphological similarity between histologies in the LNM and in the primary tumor was examined by using five to ten slides of the primary tumor. In 75 (63%) patients, the primary tumor had the same morphology in LNM. 20 (16.8%) patients had the different morphology in LNM from the primary tumor. 24 (20.2%) patients had both the same and the different morphologies in the primary tumor.

### Histologic cancer types in LNM and pathologically diagnosed differentiation in the primary tumor

In order to address that same pathologically diagnosed differentiation in the primary tumor could produce various histological types in LNM, the association between the pathologically diagnosed differentiation in the primary lesion and the histologic cancer types in LNM were compared (Table 1). The most common histologic type in the primary lesion was moderately differentiated adenocarcinoma (n = 102). In those 102 cases, the mLNs contained all tubular histology in 53, all cribriform in 27, both tubular and cribriform in 18, poorly differentiated in 2, and both cribriform and poorly differentiated in 2, indicating that different clones metastasized from the primary lesion. Similarly, in cases where the primary lesion was well differentiated, mucinous adenocarcinoma, papillary adenocarcinoma, or adenosquamous carcinoma, the histologic cancer types in the mLNs were different from those of the primary lesion.

**Table 1:** Histologic cancer types in the lymph nodes of 119 node-positive patients with colorectal cancer.

Primary lesion (Pathological diagnosis)		Lymph node (Morphological Classification)	Patients	
			*(n)*	(%)
Adenocarcinoma				
	Well differentiated	Tubular	5	4.2
		Cribriform	1	0.8
		Tubular, cribriform	2	1.7
	Moderately differentiated	Tubular	53	44.5
		Cribriform	27	22.7
		Tubular, cribriform	18	15.1
		Poorly differentiated	2	1.7
		Cribriform, poorly differentiated	2	1.7
	Poorly differentiated	Cribriform, poorly differentiated	1	0.8
Mucinous adenocarcinoma		Mucinous	3	2.5
		Tubular	2	1.7
		Cribriform	1	0.8
Papillary adenocarcinoma		Tubular	1	0.8
Adenosquamous carcinoma		Poorly differentiated	1	0.8

### CSS times by LNM histologic cancer type in primary moderately differentiated adenocarcinoma

We hypothesized that the presence of mLN with cribriform histology in other mLNs showing tubular histology suggests a malignant phenotype. We therefore compared survival times for patients with all mLNs showing tubular carcinoma, all mLNs showing cribriform carcinoma, and at least some mLNs showing either tubular or cribriform histologic types.

**Fig 3:**
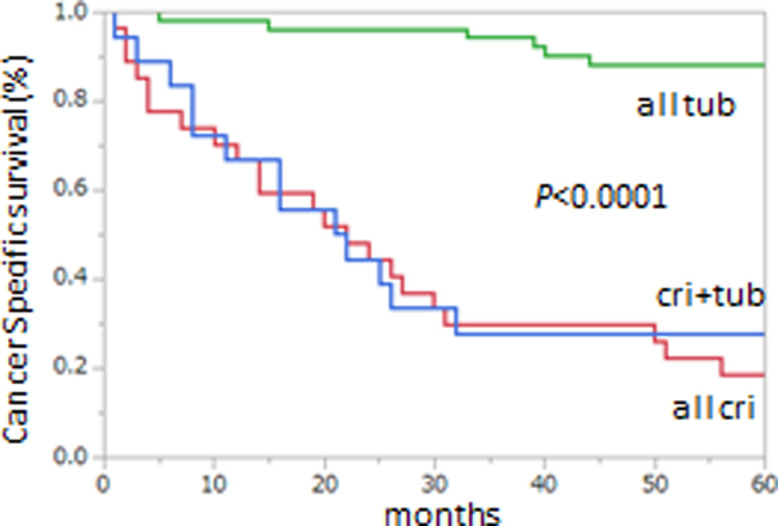
Kaplan–Meier plots of cancer-specific survival (CSS) in node-positive disease. CSS when all metastatic lymph nodes (mLNs) showed tubular carcinoma (“tub”), all mLNs showed cribriform carcinoma (“cri”), and some mLNs showed either tubular or cribriform carcinoma (“tub+cri”). All analyzed patients had a primary tumor classified as moderately differentiated adenocarcinoma.

In order to compare the histological types in LNM under the condition of the same degree of differentiation in the primary tumor, we used data only from patients with primary moderately differentiated adenocarcinoma (the most common histologic type). Kaplan–Meier plots of CSS times showed longer survival for patients with all mLNs showing tubular carcinoma than for those with all mLNs showing cribriform carcinoma or for those with at least some mLNs showing either tubular or cribriform carcinoma (p < 0.0001) (Fig 3). No survival difference was evident between patients with all mLNs showing cribriform carcinoma and with at least some mLNs showing either tubular or cribriform carcinoma (Fig 3). Those observations suggest that the presence of mLNs containing cribriform carcinoma represents a more malignant phenotype of CRC.

## Discussion

Recognition of tumor heterogeneity is important in diagnosing malignancy and in choosing treatments for cancer patients. Because a primary tumor can contain an intricate set of various cancer cell clones, distinguishing the differences is difficult. In colon cancer, LNM have been reported to be polyclonal at the genetic level [4], thus opening the possibility for distinguishing tumor heterogeneity.

There are some questions whether genetic and histological heterogeneity can exist within a single LN. In our study, each mLN contained only one histologic cancer type, and there were patients with multiple mLNs including different histologic cancer types in each mLN. The report presented that a single mLN contained a mixture of genetically different subclones derived from the primary tumor [4]. There is a possibility that the LNM may be formed by a cluster consisting of several clones, not a single clone, and that those clones with different genetic mutations may form the same histological type. Genetic analyses for our classification in LNM are needed to address this issue. Whereas, there is a report of different histological classification in LNM [9]. The report showed that LNM from CRC, within a single mLN, had homogenous histological type including either differentiated (well or moderately differentiated adenocarcinoma) or undifferentiated (mucinous or poorly differentiated adenocarcinoma) type, and heterogenous histological type including a mixture of both differentiated and undifferentiated histological types. There is no significant difference in recurrence free survival rate of patients with CRC between homogenous and heterogenous type groups. Only in CRC patients with 4 or more mLNs, homogenous histological type group was significantly worse prognosis than heterogenous type group. The report did not provide the details of histological classification for LNM. In the present study, we proposed a novel classification for LNM histology based on morphology regarding the shape of the hollow cavities and the cellular polarity. Standardization of classification for LNM histology is needed to clarify the significance.

Morphology such as lumen formation is important for understanding the malignant potential of cancer cells [10–13]. Well-differentiated adenocarcinoma usually forms tubes, lumens, or hollows. With less differentiation, no cavities are apparent. We defined four histologic cancer types in LNM from CRC. The differences between the tubular, cribriform, and poorly differentiated-types were in the shape of the empty space. “Tubular” had slender and elongated cavities. “Cribriform” had round cavities. “Poorly differentiated” had almost no hollows. We hypothesize that the smaller the empty cavity, the greater the malignancy. We previously reported that, in patients with CRC, more metastases and a poorer prognosis correlated with hollow spheroids at the invasion front rather than with tumor budding [14] or chemoresistance to 5-fluorouracil [15]. The implications of the hollow spheroid formation at the primary site might be similar to those of the cribriform histology in mLNs. To determine the shape of the cavities in detail, three-dimensional analysis will be needed. Cell polarity is also important for differentiation in cancer cells [16] and involves a variety of molecules [17–19]. Cell polarity is strong in tubular carcinoma. Molecular analysis of tubular carcinoma in mLNs might provide novel insights into cancer biology. The present report classified cancer histology in LNM from CRC, but this initial classification based on cavity presence and shape, and on cell polarity, must be refined by further investigation.

The presence of cribriform compared with tubular carcinoma in mLNs seems to suggest more malignancy. The frequency of *KRAS* mutation has been reported to be significantly higher in cribriform carcinoma than in the well-formed glandular type [20], and a higher frequency of somatic copy number alterations has been reported for cribriform carcinoma than for the well-formed glandular type [20]. Cribriform comedo-type adenocarcinoma was described as a new and distinct histologic subtype of CRC in the 2010 World Health Organization classification of tumors of the lower gastrointestinal tract [21]. Some reports have shown that cribriform carcinoma in the primary CRC was associated with lymphatic and venous invasion, metastasis to the lymph nodes, and poor survival [22,23]. In contrast, another report suggested that no prognostic differences were associated with cribriform carcinoma in the primary tumor [24]. Prognosticating for greater or lesser malignancy by detecting cribriform or tubular carcinoma in primary CRC can be difficult, because the primary tumor can contain a mixture of histologic types. In the mLNs, cancer clusters are often small and histologically uniform. Detection of histologic changes might be easier in mLNs than in the primary lesion.

Various cancer cell clones spread widely from the primary lesion. Speculation has been that “more malignant” cancer cells metastasize faster and farther than “less malignant” cancer cells. The mLNs are the front line of metastasis. The phenotype of the cancer cells in mLNs might represent the malignant potential of CRC. In the present study, CSS was significantly longer for tub group than for cri group, suggesting that when the “frontline” mLNs all show tubular carcinoma, the phenotype might be considered “less malignant.” In contrast, when “at least some” mLNs show cribriform carcinoma, the phenotype might be considered “more malignant.”

In clinical practice, after resection of the primary tumor and regional lymph nodes, patients with stage III CRC typically receive adjuvant chemotherapy based on the status of LNM [5–7]. If all mLNs show tubular carcinoma (“less malignant”), it might be possible to avoid adjuvant chemotherapy; and if “at least some” mLNs among those showing tubular carcinoma also show cribriform-type carcinoma (“more malignant”), then more aggressive therapy might be called for.

Although there is limitation with a small number of cases, we describe for reference the association between prognosis of patients with pathologically diagnosed well differentiated adenocarcinoma in the primary tumor and histologies in LNM. Of 8 cases with well differentiated adenocarcinoma in the primary tumor, 3 cases (37.5%) with cribriform type in LNM did not survive for 5 years. Whereas, one of 5 cases (20%) with tubular type in LNM did not obtain 5-year survival. Prediction of prognosis by LNM histology may be possible even in cases with well differentiated adenocarcinoma in the primary tumor. We also present for reference prognosis in patients with poorly differentiated-type in LNM. All 6 cases with at least 1 mLN showing poorly differentiated-type did not survive for 5 years. They seemed to be worse prognosis than patients with cribriform type in mLN. Poorly differentiated-type in mLN may indicate more malignant property. Large-scale investigation may clarify the implications of the histology in LNM.

Limitations of the present study include its small number of patients and retrospective nature. Further investigations, including larger retrospective studies and prospective clinical trials, are required to address the issues raised here. Moreover, a more detailed pathologic classification scheme for the histologic cancer types in the mLNs is needed to establish clinical applications.

To summarize, we demonstrated that the cancer histology in LNM from CRC was varied and could be associated with prognosis. A better understanding of cancer histology in LNM from CRC might provide novel insights in cancer research.
